# Body Adiposity Indices, Adipokines Profile, and CNR1 Polymorphisms in Atypical Phenotypes of Obesity

**DOI:** 10.3390/metabo16020091

**Published:** 2026-01-25

**Authors:** Simona Georgiana Popa, Loredana Maria Marin, Loredana Maria Dira, Ana Cristina Tudosie, Andreea Loredana Golli

**Affiliations:** 1 Department of Diabetes, Nutrition and Metabolic Diseases, University of Medicine and Pharmacy Craiova, 200349 Craiova, Romania; simona.popa@umfcv.ro; 2 Department of Pharmaceutical Physics, Faculty of Pharmacy, “Carol Davila” University of Medicine and Pharmacy, 020956 Bucharest, Romania; 3 Department of Pediatrics, University of Medicine and Pharmacy, 200349 Craiova, Romania; maria.dragan@umfcv.ro; 4 Department of Endocrinology, University of Medicine and Pharmacy, 200349 Craiova, Romania; ana.dragomirescu@umfcv.ro; 5 Department of Public Health and Healthcare Management, University of Medicine and Pharmacy, 200349 Craiova, Romania; andreea.golli@umfcv.ro

**Keywords:** Insulin-Resistant Normal Weight phenotype, Insulin-Sensitive Obesity phenotype, adipocytokines, cannabinoid receptor type 1 gene polymorphisms, body adiposity indices

## Abstract

**Background/Objectives:** Insulin-Resistant Normal Weight and Insulin-Sensitive Obesity are atypical cardiometabolic phenotypes whose clinico-biological features, management, and prognosis are a subject of extensive scientific debate. The current study aimed to assess the prevalence of metabolic phenotypes of obesity and to evaluate their association with markers related to diabesity, adipokines profile, and two single nucleotide polymorphisms of CNR1 gene. **Methods:** We performed a cross-sectional analysis in a random sample of 487 individuals (53.03 ± 13.71 years, 48.3% male) which were classified based on body mass index (</≥25 kg/m^2^) and insulin resistance (HOMA-IR cut-off value 2.5) as Insulin-Sensitive/Insulin-Resistant Normal Weight (ISNW/IRNW) and Insulin-Sensitive/Insulin-Resistant Obesity (ISO/IRO). **Results:** The ISO phenotype frequency was 24.2%, with a higher prevalence in the 40–60 years age group (47.0%) and in men (44.9%), while the prevalence of IRNW was 7.0%, predominating in women (61.8%). Participants with IRNW had a more altered glycoregulation profile (fasting and 2 h OGTT blood glucose, prediabetes, and hyperinsulinism), hypercholesterolemia, and adiposity indices (ABSI) than those with ISNW, but comparable to those with IRO. Participants with ISO had a more favorable glycoregulation profile, lipid profile, adipocytokines, and adiposity indices than those with IRO. IRNW had higher odds of being associated with prediabetes (OR 10.75; *p* < 0.001) than ISNW, while younger age, CUN-BAE, and ABSI were independently associated with both ISO and IRNW phenotypes. **Conclusions:** The IRNW phenotype should be actively evaluated to intervene on the cardiometabolic risk, while further studies are needed to confirm the sustainability of the favorable cardiometabolic profile of the ISO phenotype.

## 1. Introduction

Obesity and insulin resistance represent major global public health challenges in the context of rising morbidity worldwide. Therefore, the clinical importance of obesity and insulin resistance should not be underestimated, considering the mortality and morbidity associated with the multiple conditions that constitute their consequences [1,2,3,4,5,6].

Globally, obesity prevalence continues to rise. In 2022, an estimated 2.5 billion adults were classified as overweight, of whom 890 million were living with obesity, while approximately 390 million children and adolescents aged 5–19 years were overweight, including 160 million affected by obesity [7]. It is predicted that by 2050 approximately 3.8 billion adults will be overweight or obese, up from roughly 1 billion in 2021, reflecting a continuous increase across all regions [8]. In 2022, the prevalence of obesity within the European Union showed substantial variation across member states, ranging between 6.1% in Italy and 28.7% in Malta. Overall, in most countries, the proportion of obese men exceeded that of women [9]. In Romania, the PREDATORR study reported the following high prevalence rates: 31.9% of adults were obese, 34.7% were overweight, 73.9% had abdominal obesity, and 38.5% had metabolic syndrome. These values surpass the European average for obesity (~16.7%) and the worldwide estimate of 13% [1]. More recent national estimates from a 2022 Romanian survey place adult obesity at 22.5% (range 18.3–29%) and project it could reach 35% by 2035 [10].

Therefore, obesity is a growing public health problem that leads to significant mortality and morbidity worldwide [3,11]. The cluster of obesity, insulin resistance, dyslipidemia, and type 2 diabetes mellitus is a polygenic multifactorial condition in which the phenotype is the result of the interaction between environmental factors and genetic predisposition. However, not all obese individuals present metabolic risk factors and also not all normal-weight individuals are metabolically healthy. This has led to the identification of different metabolic phenotypes of obesity that represent different subgroups characterized by specific clinical and biological features, which may influence the response to treatments and long-term prognosis: Metabolically Unhealthy Lean or Insulin-Resistant Normal Weight (MUHL/IRNW), Metabolically Healthy Obese or Insulin-Sensitive Obesity (MHO/ISO), Metabolically Healthy Lean or Insulin-Sensitive Normal Weight (MHL/ISNW), and Metabolically Unhealthy Obese/Insulin-Resistant Obesity (MUHO/IRO) [1,2,3,6,11,12,13,14,15]. This classification suggests that excess body fat does not always indicate metabolic dysfunction, highlighting the complex relationship between adiposity and metabolic health.

IRNW individuals exhibit adverse metabolic profiles, including hypertriglycer-idemia, low HDL cholesterol, prediabetes, or diabetes, resulting in higher odds of abdominal obesity, impaired kidney function, and a 10-year Framingham-predicted cardiovascular risk (OR 5.8) compared with ISNW [1]. ISO participants exhibit elevated BMI or waist circumference but maintain normal renal function, lower rates of hypertension, diabetes, and dyslipidemia, and do not exhibit the elevated cardiovascular risk associated with IRO. However, ISO participants are still associated with certain metabolic issues when compared to the ISNW group, including hypo-HDL cholesterolemia and prediabetes [1,2,3]. Consequently, ISO is characterized by preserved insulin sensitivity, lower homeostasis model assessment for insulin resistance (HOMA-IR), appropriate beta- and alpha-cell function, and balanced incretin responses, which collectively support glucose homeostasis despite excess adiposity [3]. In contrast, IRO combines obesity with pronounced metabolic disturbances, including hyperinsulinemia, dyslipidemia, systemic inflammation, and the highest risk for cardiovascular disease, type 2 diabetes, and renal impairment [2,3]. This classification emphasizes that metabolic health is not only determined by BMI, highlighting the heterogeneity of obesity and the importance of integrating metabolic and anthropometric parameters to identify high-risk individuals.

Adipokines, such as adiponectin and leptin, are essential for glucose metabolism and energy balance [16]. High adiponectin levels are associated with anti-inflammatory and insulin-sensitizing effects, while elevated leptin levels reflect greater fat mass [11,16]. Therefore, the ratio between adiponectin and leptin may serve as an indicator of metabolic health and help distinguish between metabolically healthy and unhealthy obesity.

Another significant factor influencing metabolic risk is the distribution of fat. Visceral adiposity is more harmful than subcutaneous fat, being closely linked to insulin resistance and chronic low-grade inflammation [12]. To better evaluate this distribution, several indices have been developed, such as the visceral adiposity index (VAI), lipid accumulation product (LAP), conicity index (CI), body shape index (ABSI), and Clinica Universidad de Navarra Body Adiposity Estimator (CUN-BAE) [13,17,18,19]. These indices complement traditional measures such as BMI and waist circumference, offering a more detailed assessment of body composition and its metabolic consequences. Their combined use can provide additional information about adipose tissue function and its contribution to metabolic risk [17,18,19].

Genetic factors also contribute to the variability of obesity phenotypes. Appetite, lipid metabolism, and energy balance are all significantly regulated by the endocannabinoid system. It includes endogenous ligands and receptors, such as the cannabinoid receptor type 1, encoded by the cannabinoid receptor type 1 (CNR1) gene. Overactivation of this receptor promotes increased food intake, fat accumulation, and insulin resistance. Genetic polymorphisms of CNR1 can influence how the endocannabinoid system functions, which may help explain why some individuals with obesity remain metabolically healthy while others develop metabolic syndrome [4,11,16,20]. Variants such as rs806368, rs1049353, rs12720071, rs806381, rs10485170, and rs6454674 have been linked to differences in fat distribution, adipokine secretion, and glucose metabolism [4,5,16,21].

Understanding how adipokines, body adiposity indices, and CNR1 polymorphisms interact could clarify the mechanisms behind atypical obesity phenotypes, including ISNW, IRNW, ISO, and IRO. These categories reflect the diversity of metabolic responses to similar degrees of adiposity. Identifying the biological and genetic factors that define these phenotypes may help predict which individuals are more likely to develop metabolic disease.

The current study aimed to assess the prevalence of different metabolic phenotypes of obesity and to evaluate their association with markers related to diabesity, adipokines profile, and two single nucleotide polymorphisms of CNR1 gene.

## 2. Materials and Methods

### 2.1. Study Population

Subjects were recruited from the database of National Council of Scientific Research in Higher Education, PN II Program: IDEAS—Exploratory Research Projects grant number 234/2007 “New perspectives in the study of endocannabinoids—CNR1 Polymorphism and metabolic phenotype”. The purpose of the study was to define classical and genetic risk factors of obesity metabolic phenotypes in Caucasian with Romanian ancestral origin, unrelated adults (aged 35–75 years). The participants were randomly recruited between September 2007 and September 2008, from the Specialized Outpatient Clinic for subjects with type 2 diabetes or prediabetes and obesity/overweight and from the population of Craiova by distributing informative materials. All the subjects were evaluated, and if they met the inclusion criteria, they were classified according to metabolic phenotype. Exclusion criteria were as follows: diagnosis of type 1 diabetes, conditions (secondary or gestational diabetes or diseases, except type 2 diabetes/prediabetes, associated with insulin resistance), or medications that interfere with insulin secretion and insulin resistance, pregnancy, and lactation. The study has been designed to comply with the Declaration of Helsinki, and the protocol has been approved by the Institutional review board of National Council of Scientific Research in Higher Education, PN II Program: IDEAS—Exploratory Research Projects under contract No. 234/01.10.2007, when the project was accepted for funding and the informed consent was obtained from all individuals enrolled in the study.

### 2.2. Clinical and Laboratory Data

A trained interviewer collected from all participants the sociodemographic information, including age, smoking status, medication history, past medical history, and family history of obesity, using questionnaires. The physical examination included anthropometric measurements and blood pressure evaluation, using standard procedures. Participants with BMI 25–30 kg/m^2^ were categorized as being overweight and those with BMI ≥ 30 kg/m^2^ were considered as being obese. Abdominal obesity, hypertension, and metabolic syndrome were defined according to Harmonizing the metabolic syndrome: a joint interim statement of the International Diabetes Federation Task Force on Epidemiology and Prevention; National Heart, Lung, and Blood Institute; American Heart Association; World Heart Federation; International Atherosclerosis Society; and International Association for the Study of Obesity [15].

Venous blood samples were collected after 12 h of fasting and plasma levels of glucose, triglycerides, and total cholesterol were evaluated using enzymatic method; HDL cholesterol by precipitation method and insulin; and leptin and adiponectin were assessed by ELISA test with monoclonal antibodies. LDL cholesterol was calculated using the Friedewald equation. Insulin resistance (HOMA-IR) and insulin secretion (HOMA-%B) were estimated based on fasting blood glucose and insulin levels, using the following formulas [22]:

HOMA-IR = [fasting insulinemia (µU/mL) × fasting blood glucose (mmol/L)]/22.5

The HOMA-IR value of 2.5 was considered as the threshold value for defining insulin resistance.

HOMA-% B = [fasting insulinemia (µU/mL) × 20]/[fasting blood glucose (mmol/L) − 3.5]

Body adiposity indices (CUN-BAE, LAP, VAI, ABSI, and CI) were calculated using the following formulas [13,17,18,19]:

**VAI** = Waist/(39.68 + 1.88 × BMI) × (TG/1.03) × (1.31/HDL Cholesterol)

in male and

**VAI** = Waist/(39.58 + 1.89 × BMI) × (TG/0.81) × (1.52/HDL Cholesterol)

in female; BMI in kg/m^2^, waist in centimeters and TG and HDL cholesterol in mmol/L

**LAP** = (Waist − 65) × TG

in male and

**LAP** = (Waist − 58) × TG

in female; waist in centimeters and TG in mmol/L

**CUN-BAE** = −44.98 + (0.503 × Age) + (10.689 × Gender) + (3.172 × BMI) − (0.026 × BMI^2^) + (0.181 × BMI × Gender) − (0.02 × BMI × Age) − (0.005 × BMI^2^ × Gender) + (0.00021 × BMI^2^ × Age),

where female = 1 and male = 0; age in years, BMI in kg/m^2^

**CI** = Waist/{0.109 × √(Weight/Height)};

weight in kg, waist and height in meters

**ABSI** = Waist/(BMI^2/3^ × Height^1/2^);

BMI in kg/m^2^, waist and height in meters

Genomic DNA was prepared from leukocytes using standard methods. The CNR1 single nucleotide polymorphisms—rs754387 and rs806368—were genotyped by TaqMan probe-based Real-Time Polymerase Chain Reaction assays (Corbett Rotor-Gene 6000) performed under standard conditions.

Standard oral glucose tolerance test (OGTT) was performed in all subjects who had no personal pathological history of diabetes.

Hypertriglyceridemia was defined as TG ≥ 150 mg/dL and hypo-HDL cholesterolemia was defined as HDL levels < 40 mg/dL in men or <50 mg/dL in women or drug treatment for reduced HDL or drug treatment for dyslipidemia [15]. Hypercholesterolemia was considered when TC ≥ 200 mg/dL and hyper-LDL cholesterolemia was considered when LDL ≥ 100 mg/dL and/or statin therapy was used [23].

Based on the HOMA-IR, the cut-off value for insulin resistance being 2.5 and BMI, and the cut-off value for overweight/obesity being 25 kg/m^2^, the following four metabolic phenotypes were defined: ISNW Insulin-Sensitive Normal Weight (HOMA-IR < 2.5 and BMI < 25 kg/m^2^), IRNW Insulin-Resistant Normal Weight (HOMA-IR ≥ 2.5 and BMI < 25 kg/m^2^), ISO Insulin-Sensitive Obesity (HOMA-IR < 2.5 and BMI ≥ 25 kg/m^2^), and IRO Insulin-Resistant Obesity (HOMA-IR ≥ 2.5 and BMI ≥ 25 kg/m^2^).

### 2.3. Statistical Analysis

Continuous variables were expressed as mean and standard deviation, and categorical variables were expressed as percentages. Body adiposity indices, leptin, and adiponectin were categorized into tertiles to analyze their relationships, since there are no established threshold values for defining pathology.

Non-parametric tests (Mann–Whitney U and Chi-squared) were used for comparisons between metabolic phenotypes of continuous and categorical variables. Multinomial logistic regression was performed to assess the association of anthropometric, metabolic, demographic, and CNR1 polymorphisms (independent variables) with metabolic phenotypes (dependent variable), after adjusting for covariates (sex, smoking), and ISNW being considered reference category. *p* < 0.05 (two-tailed) was considered significant. Analyses were performed using SPSS software v19.0.

## 3. Results

A total of 487 individuals were included in this cross-sectional study, with a mean age of 53.03 ± 13.71 years, and 48.3% were male. The groups with the two atypical metabolic phenotypes—IRNW and ISO—comprised 7.0% (34 subjects) and 24.2% (118 subjects) of the sample, respectively (Table 1).

The baseline characteristics of the participants compared among the four obesity phenotypes metabolically are presented in Table 2.

Compared to the control group of ISNW individuals, participants with ISO were older, predominantly over 40 years of age, and had a more frequent family history of obesity.

Participants with ISO were older, with a predominance of age over 40 years, both compared to the control group of ISNW individuals, but also to those with IRO, indicating that there would have been enough time to develop an unhealthy metabolic profile associated with obesity.

Also, although the frequency of a family history of obesity was higher in ISO compared to the control group, there were no significant differences from IRO both in terms of family history of obesity and in terms of the frequency of CNR1 gene polymorphisms, indicating a less important role of genetic predisposition in the occurrence of the pathological metabolic profile.

In participants with ISO, overweight prevailed (64.4%), while in those with IRO, abdominal obesity and BMI > 30 kg/m^2^ were more frequent, which could explain the more favorable metabolic profile of those with ISO (Table 2).

Although IRNW had age, family history of obesity, and waist circumference values comparable to ISNW, they presented abdominal obesity more frequently (28.40% in ISNW vs. 50.0% in IRNW), which may justify an unhealthy metabolic profile characterized by fasting and 2 h hyperglycemia during OGTT, prediabetes, and hypercholesterolemia, compared to ISNW and similar to that of IRO participants (Table 2).

Also, although compared to IRO, participants with IRNW have significantly less frequent abdominal obesity, a hereditary and personal history of pathological obesity, and are predominantly older than 60 years of age; they have an unhealthy metabolic profile, comparable to IRO, despite being normal weight (Table 2).

Participants with ISO generally have a more favorable metabolic profile compared to IRO and less favorable than those with ISNW, except for insulin secretion which does not differ significantly between ISO and ISNW and is significantly lower in ISO compared to IRO (HOMA-%B 64.92 ± 58.00 in ISNW vs. 68.18 ± 53.32 in ISO vs. 112.02 ± 73.93 in IRO) (Table 3).

The leptin level, significantly higher in those with ISO compared to ISNW and lower compared to IRO (7.71 ± 11.75 in ISNW vs. 13.93 ± 11.91 in ISNW vs. 20.8 ± 24.32 in IRO), may indicate leptin resistance associated with insulin resistance, the differences being explained by the fact that overweight predominates in those with ISO and BMI > 30 kg/m^2^ predominates in those with IRO (Table 3). Leptin levels may explain the better metabolic profile of those with ISO compared to those with IRO, although excess weight is present in both categories (Table 3).

Participants with IRNW had a much higher insulin secretion compared to those with ISNW, possibly secondary to insulin resistance, although they did not have a higher frequency of diabetes mellitus compared to ISNW. Insulin secretion of IRNW was comparable to that of IRO although they had a lower degree of insulin resistance and no significant differences were detected in terms of glycoregulation disorders.

In both individuals with IRNW and those with IRO, hyperleptinemia prevailed (frequency of the 3rd tertile of leptin 9.3% in ISNW vs. 26.5% in IRNW vs. 42.3% in IRO), compared to the control group. All adiposity indices had comparable values in those with IRNW and ISNW, except CI and ABSI, which were significantly higher in IRNW compared to ISNW (Table 3).

Genetic analysis for the CNR1 rs754387 polymorphism was performed in 384 subjects. The CC genotype was found in 41.15%, AC in 58.07%, and AA in 0.78% of the individuals. The frequency of the C allele was *p* = 0.702 and of the A allele, q = 0.298. The expected genotype frequencies (Hardy–Weinberg) were f(CC) = 0.4926, f(CA) = 0.4185, and f(AA) = 0.0889. Because the AA genotype was present in <5% of the individuals, the analysis was performed according to the C dominant model (AC + AA vs. CC).

Genetic analysis for the CNR1 rs806368 polymorphism was performed in 403 subjects. The TT genotype was found in 56.58%, TC in 42.18%, and CC in 1.24% of the individuals. The frequency of the T allele was *p* = 0.777 and of the C allele, q = 0.223. The expected genotype frequencies (Hardy–Weinberg) were f(TT) = 0.6032, f(TC) = 0.3469, and f(CC) = 0.0499. Because the CC genotype was present in <5% of the individuals, the analysis was performed according to the T dominant model (TC + CC vs. TT).

Multivariate multinomial logistic regression analysis showed that the IRNW phenotype was associated with prediabetes (*p* = 0.009) and the third tertile (≥0.083) of ABSI (*p* = 0.045) (Table 4). Prediabetes was also independently associated with IRO phenotype (Table 4). Both the IRNW and ISO phenotypes had higher odds of being associated with younger age (β = −0.106, *p* = 0.001 for IRNW, and β = −0.063, *p* = 0.037 for ISO) and higher CUN-BAE value (*p* = 0.035 for IRNW and *p* < 0.001 for ISO) (Table 4).

## 4. Discussion

The study offers an extensive evaluation of metabolic phenotypes of obesity, combining anthropometric indices, adipokine profiles, and CNR1 gene polymorphisms in a Romanian adult population. Our analysis provides a better knowledge of the variety of obesity and its metabolic consequences by simultaneously evaluating body composition indicators, genetic factors, and conventional metabolic markers. The results support the evidence that obesity should not be viewed as a single, uniform disorder but rather as a spectrum of distinct metabolic phenotypes, each carrying its own level of cardiometabolic risk.

### 4.1. Heterogeneity of Metabolic Phenotypes

Although prevalence varies depending on ethnicity, lifestyle, and diagnostic crite-ria, the distribution of metabolic phenotypes identified in this cohort is similar to that identified in other studies, with roughly half of the participants classified as IRO, followed by ISO, ISNW, and IRNW [1,2,3]. The analysis of differences in the prevalence of metabolic phenotypes of obesity reported in different studies is difficult to achieve due to the fact that the demographic characteristics of the populations analyzed differ (children, postmenopausal women, elderly, etc.), and the different periods of study development due to the evolution of obesity/insulin resistance therapies influence the prevalence of obesity phenotypes. Also, the prevalence of metabolic phenotypes cannot be compared because there is no harmonized definition of metabolic phenotypes of obesity, in some studies these being defined based on the presence of metabolic syndrome, in others based on different surrogate markers of insulin resistance—HOMA-IR, eGDR, etc. n [1,2,3,6,11,12,14,19,24]. The relatively high proportion of ISO individuals in our study (24.2%) emphasizes that a considerable part of obese people can maintain preserved insulin sensitivity and a favorable lipid profile, despite elevated body mass index and waist circumference. On the other hand, a significant percentage of lean people who are metabolically sick (7%) shows that normal weight does not ensure metabolic health. These results confirm that BMI alone cannot reflect the true metabolic risk of obesity, underscoring the value of combining biochemical and body composition measures for accurate risk assessment.

Regarding the differences in insulin secretion found between ISO vs. IRO and ISNW, the potential explanations is that in those with ISNW and ISO insulin resistance is not so high as to induce secondary hyperinsulinism, as is the case in those with IRO, and also that in those with ISO, diabetes, which involves an insulin secretory deficit, is predominant (43.2%), whereas in those with IRO, prediabetes predominates (47.0%).

### 4.2. Adipokines and Metabolic Health

Adiponectin and leptin were found to be significant differentiators among the metabolic phenotypes. IRO individuals presented the lowest adiponectin levels and the highest leptin concentrations, confirming a state of adipose tissue dysfunction, low-grade inflammation, and insulin resistance [11,24,25,26]. In contrast, ISO individuals had comparatively higher levels of adiponectin and lower levels of leptin, indicating that adipocyte function and anti-inflammatory signaling were maintained. These findings reinforce the hypothesis that metabolic health is not solely dictated by body weight but by a complex interplay between adipokine dysregulation, insulin resistance, and genetic background. Our findings are consistent with earlier studies, which demonstrated that even in overweight people, a greater adiponectin to leptin ratio is associated with better insulin sensitivity and lower cardiometabolic risk [25,26,27].

Adiponectin’s insulin-sensitizing and anti-atherogenic properties, together with leptin’s role in appetite regulation and energy consumption, create a dynamic endocrine balance that determines whether excess adiposity leads to metabolic dysfunction [28,29]. Therefore, the difference observed between the IRO and IRNW groups may reflect a link between disrupted adipose tissue signaling and the development of diabesity-related complications. These findings are consistent with studies indicating that hyperleptinemia and hypoadiponectinemia are characteristics of the metabolically unhealthy state, independent of BMI or waist circumference [11,24].

### 4.3. Adiposity Indices as Metabolic Markers

Beyond standard body measurements, the adiposity indices analyzed, including LAP, VAI, CI, ABSI, and CUN-BAE, offered additional insight into how fat distribution relates to metabolic health. The LAP and CI were significantly higher in IRO participants and showed strong correlations with markers of insulin resistance, highlighting their usefulness in detecting visceral obesity and related cardiometabolic risks [30,31,32]. These results support earlier studies demonstrating that the main causes of metabolic syndrome and type 2 diabetes are lipid accumulation and central adiposity rather than total fat mass [6,11,33].

Comparable VAI and ABSI values in participants with ISO and ISNW associated with significantly higher CUN-BAE values in ISO compared to ISNW indicate that those with ISO have a favorable metabolic and lipid profile, despite an increased BMI. The same is indicated by the fact that CUN-BAE values are comparable in those with ISO and IRO, but the values of body adiposity indices that include lipid parameters are significantly lower in those with ISO compared to those with IRO.

Small but consistent differences in CI and ABSI values distinguished insulin-sensitive from insulin-resistant individuals, indicating that subtle variations in body fat distribution are associated with metabolic risk. Overall, these findings emphasize the value of using multiple adiposity indices together to achieve a more complete assessment of metabolic health, an approach increasingly encouraged in both clinical and epidemiological research.

### 4.4. Role of CNR1 Polymorphisms

In the genetic component of the study, no significant associations were found between the two CNR1 single nucleotide polymorphisms (rs806368 and rs754387) and the distribution of metabolic phenotypes. This observation suggests that, within this Romanian cohort, these variants might only have a minor impact on metabolic profile differentiation. However, the endocannabinoid system, in which the CNR1 gene plays a central role, remains an important biological pathway that connects appetite regulation, lipid metabolism, and insulin sensitivity [16,34]. Overactivation of the cannabinoid receptor type 1 has been implicated in increased energy intake, adipogenesis, and dyslipidemia [35,36,37]. Previous studies identified other CNR1 polymorphisms, such as rs1049353 and rs1049353, as potential modulators of obesity-related traits, but their effects appear to vary by ethnicity and environmental exposures [16,38]. Therefore, while our results do not confirm a direct genetic effect, they underscore the need for larger, multi-ethnic studies and genome-wide analyses to clarify how CNR1 genetic variability interacts with adipokine signaling and body fat distribution.

### 4.5. Integrative Interpretation

The coexistence of insulin-sensitive and -resistant forms of both obesity and leanness highlight the complexity of metabolic health. It likely results from the interaction of genetic predisposition, adipose tissue expandability, adipokine secretion, and environmental influences such as diet, physical activity, and psychosocial stress.

Clinically, the predominance of IRO (50.7%) and the relative rarity of IRNW (7.0%) emphasize that the main metabolic burden arises from excess adiposity combined with insulin resistance. At the same time, the presence of ISO individuals (24.2%) demonstrates that preserved metabolic function can coexist with obesity, although this condition may be transient.

The identification of individuals with ISO or IRNW phenotypes has important clinical implications, as these groups may benefit from early lifestyle or pharmacological therapies based on their metabolic profile rather than just body weight. Also, considering the cardiometabolic particularities of obesity, it is important to monitor the therapeutic success of pharmacological interventions or bariatric surgery, not only from an anthropometric perspective but also from a cardiometabolic parameters perspective [39]. Furthermore, evidence suggests that the ISO phenotype is not a stable condition and not free from cardiovascular disease risk, since a significant proportion of such individuals eventually develop metabolically unhealthy obesity, emphasizing the need of continuous monitoring and preventive strategies [40].

### 4.6. Clinical and Research Implications

From a clinical perspective, our findings highlight the importance of incorporating simple yet powerful adiposity indices (such as LAP, CI, and CUN-BAE) and adipokine measurements into the routine evaluation of individuals at risk for metabolic disease. This strategy can improve early detection of metabolic dysfunction, guide personalized interventions, and reduce the burden of diabesity in the general population. In research contexts, the observed independence between BMI and metabolic health emphasizes the necessity for advanced phenotyping methods in obesity-related studies. This multidimensional approach, integrating metabolic, hormonal, and genetic factors, represents a promising direction for precision medicine in endocrinology and metabolic research.

The limitation of the current study is mainly represented by its cross-sectional design, which does not allow the analysis of the evolution of the cardiometabolic profile of the two atypical phenotypes—ISO and IRNW.

## 5. Conclusions

The IRNW atypical phenotype should be actively detected to intervene on the cardiometabolic risk of subjects with normal weight, while the ISO phenotype should be followed over time to observe whether it is only an early stage of obesity evolution or a stable favorable cardiometabolic phenotype.

Future research should focus on a harmonized definition and on the cardiometabolic risks of the two atypical phenotypes—IRNW and ISO—so that adapted therapeutic and preventive interventions can be implemented.

## Figures and Tables

**Table 1:** Frequency of insulin resistance, overweight/obesity, and metabolic phenotypes of obesity.

Parameters	Categories	Prevalence n (%)
HOMA-IR	<2.5	206 (42.3%)
	≥2.5	281 (57.7%)
BMI	<25 kg/m^2^	122 (25.1%)
	≥25 kg/m^2^	365 (74.9%)
Metabolic phenotypes of obesity	ISNW	88 (18.1%)
	IRNW	34 (7.0%)
	ISO	118 (24.2%)
	IRO	247 (50.7%)

HOMA-IR homeostasis model assessment for insulin resistance, BMI body mass index, ISNW Insulin-Sensitive Normal Weight (HOMA-IR < 2.5 and BMI < 25 kg/m^2^), IRNW Insulin-Resistant Normal Weight IRNW (HOMA-IR ≥ 2.5 and BMI < 25 kg/m^2^), ISO Insulin-Sensitive Obesity ISO (HOMA-IR < 2.5 and BMI ≥ 25 kg/m^2^), and IRO Insulin-Resistant Obesity IRO(HOMA-IR ≥ 2.5 and BMI ≥ 25 kg/m^2^).

**Table 2:** Clinical and biological characteristics according to obesity metabolic phenotypes.

Variables		ISNW	ISO	IRNW	IRO	Overall
Gender (male), %		47.7	44.9	38.2	51.4	48.3
Age (years), mean (SD)		49.72 ± 17.34	55.85 ± 12.54 ^a,e^	49.03 ± 17.82	53.43 ± 11.73	53.03 ± 13.71
Age groups, %	Overall		^a,d^	^f^	^c^	
	<40 years	35.2	14.5	35.3	15.4	20.2
	40–60 years	35.2	47.0	23.5	53.4	46.5
	>60 years	29.5	38.5	41.2	31.2	33.3
Currently smoking/past smoker, %		38.7	26.8	44.4	36.4	34.7
BMI (kg/m^2^), mean (SD)		22.08 ± 2.14	29.24 ± 3.6 ^a,d,e^	22.45 ± 2.32 ^f^	31.3 ± 4.92 ^c^	28.52 ± 5.54
BMI categories	Overall		^a,d,e^	^f^	^c^	
	<25 kg/m^2^	100.0	0.0	100.0	0.0	25.9
	25–30 kg/m^2^	0.0	64.4	0.0	47.4	38.8
	≥30 kg/m^2^	0.0	35.6	0.0	52.6	35.3
Maximum BMI (kg/m^2^), mean (SD)		23.9 ± 2.77	30.65 ± 4.03 ^a,d,e^	24.69 ± 3.62 ^f^	32.9 ± 5.42 ^c^	30.16 ± 5.84
Maximum BMI categories	Overall		^a,d,e^	^f^	^c^	
	<25 kg/m^2^	71.6	1.7	58.8	0.8	17.9
	25–30 kg/m^2^	26.1	50.8	29.4	34.0	36.3
	≥30 kg/m^2^	2.3	47.5	11.8	65.2	45.8
Family history of obesity, (%)		17.2	34.7 ^a,d^	17.6 ^f^	42.3 ^c^	34.2
Waist (cm), mean (SD)		81.08 ± 11	97.6 ± 10.4 ^a,d,e^	84.71 ± 11.13 ^f^	104.75 ± 11.71 ^c^	97.34 ± 14.58
Abdominal obesity, %		28.40	92.40 ^a,d,e^	50.00 ^b,f^	97.20 ^c^	80.3
FPG (mg/dL), mean (SD)		103.61 ± 26.29	107.35 ± 16.59 ^a,e^	132.37 ± 78.17 ^b^	124.28 ± 36.97 ^c^	117.01 ± 37.35
1 h OGTT Glycemia (mg/dL), mean (SD)		160.46 ± 57.84	174.08 ± 58.46 ^e^	178.87 ± 58.18	201.69 ± 62.41 ^c^	185.26 ± 62.4
2 h OGTT Glycemia (mg/dL), mean (SD)		114.11 ± 36.92	139.22 ± 53.73 ^a,e^	140.66 ± 63.88 ^b^	157.8 ± 63.45 ^c^	143.53 ± 58.96
Glucose tolerance	Overall		^a,e^	^b^	^c^	
	NGT	50	27.1	26.5	13.4	24.2
	Prediabetes	19.3	29.7	38.2	47.0	37.2
	Diabetes	30.7	43.2	35.3	39.7	38.6
TC (mg/dL), mean (SD)		175.63 ± 39.14	187.46 ± 43.27	191.15 ± 40.1	193.28 ± 45.22 ^c^	188.53 ± 43.72
Hypercholesterolemia, %		37.5	51.3 ^a,e^	58.8 ^b^	63.8 ^c^	55.7
TG (mg/dL), mean (SD)		122.65 ± 87.92	124.93 ± 73.27 ^e^	134.01 ± 103.13 ^f^	157.56 ± 100.62 ^c^	141.74 ± 93.78
Hypertriglyceridemia, %		37.5	44.4 ^e^	41.2 ^f^	66.3 ^c^	54.0
HDL cholesterol (mg/dL), mean (SD)		52.34 ± 15.86	51.98 ± 15.94 ^e^	51.56 ± 16.5 ^f^	46.68 ± 15.71 ^c^	49.32 ± 16.02
Hypo-HDL cholesterolemia, %		36.4	46.2 ^e^	38.2 ^f^	56.7 ^c^	49.2
LDL cholesterol (mg/dL), mean (SD)		98.75 ± 35.15	110.5 ± 39.44	112.79 ± 30.35	115.08 ± 40.44 ^c^	110.86 ± 39.01
Hyper-LDL cholesterolemia, %		61.4	67.5 ^e^	64.7	76.8 ^c^	70.9
Hypertension, %		35.2	50.0 ^a^	38.2 ^f^	59.1 ^c^	51.1
Metabolic syndrome (yes), %		23.9	66.7 ^a,d,e^	41.2 ^b,f^	84.2 ^c^	66.0

BMI body mass index, ISNW Insulin-Sensitive Normal Weight, IRNW Insulin-Resistant Normal Weight, ISO Insulin-Sensitive Obesity, IRO Insulin-Resistant Obesity, OGTT oral glucose tolerance test, FPG fasting plasma glucose, NGT normal glucose tolerance, TC total cholesterol, TG triglycerides, HDL high-density lipoprotein, LDL low-density lipoprotein, and SD standard deviation; ^a^ *p* < 0.05 for ISO vs. ISNW; ^b^ *p* < 0.05 for IRNW vs. ISNW; ^c^ *p* < 0.05 for IRO vs. ISNW; ^d^ *p* < 0.05 for IRNW vs. ISO; ^e^ *p* < 0.05 for IRO vs. ISO; and ^f^ *p* < 0.05 for IRO vs. IRNW.

**Table 3:** Adipocytokines, body adiposity indices, and CNR1 gene polymorphism according to obesity metabolic phenotypes.

Variables			ISNW	ISO	IRNW	IRO	Overall
Adiponectin (ng/mL), mean (SD)			13.94 ± 12.13	11 ± 7.32	14.33 ± 16.77	10.21 ± 9.8 ^c^	11.34 ± 10.5
Adiponectin tertiles, %		Overall				^c^	
		<5.9	22.1	33.7	35.5	36.5	
		5.9–12.5	36.8	28.4	19.4	36.5	
		≥12.5	41.2	37.9	45.2	27.0	
Leptin (ng/mL), mean (SD)			7.71 ± 11.75	13.93 ± 11.91 ^a,e^	10.28 ± 10.84 ^f^	20.8 ± 24.32 ^c^	16.06 ± 19.85
Leptin tertiles, %		Overall		^a,e^	^b,f^	^c^	
		<5.7	64.0	32.2	55.9	19.9	
		5.7–17.8	26.7	33.9	17.6	37.8	
		≥17.8	9.3	33.9	26.5	42.3	
Insulin (µU/mL), mean (SD)			5.45 ± 2.61	6.56 ± 2.19 ^a,d,e^	13.3 ± 5.3 ^b,f^	15.37 ± 6.73 ^c^	11.3 ± 6.9
HOMA-IR, mean (SD)			1.36 ± 0.63	1.72 ± 0.58 ^a,d,e^	3.9 ± 1.32 ^b,f^	4.64 ± 2.4 ^c^	3.29 ± 2.33
HOMA-% B, mean (SD)			64.92 ± 58.00	68.18 ± 53.32 ^d,e^	124.58 ± 114.38 ^b^	112.02 ± 73.93 ^c^	93.82 ± 74.09
VAI, mean (SD)			1.80 ± 1.88	1.87 ± 1.30 ^e^	2.20 ± 2.09 ^f^	2.70 ± 2.28 ^c^	2.30 ± 2.03
VAI tertiles, %		Overall		^e^	^f^	^c^	
		<1.34	51.1	43.6	52.9	19.4	
		1.34–2.28	31.8	32.5	17.6	35.6	
		≥2.28	17.0	23.9	29.4	44.9	
LAP, mean (SD)			30.13 ± 32.9	51.42 ± 32.79 ^a,d,e^	39.25 ± 40.41 ^f^	78.75 ± 57.50 ^c^	60.6 ± 51.26
LAP tertiles, %		Overall		^a,e^	^f^	^c^	
		<33.31	69.3	37.3	52.9	16.6	
		33.31–66.48	20.5	39.8	32.4	34.4	
		≥66.48	10.2	22.9	14.7	49.0	
CUN-BAE, mean (SD)			26.69 ± 6.32	37.04 ± 7.71 ^a,d^	28.29 ± 7.19 ^f^	38.37 ± 7.82 ^c^	35.25 ± 8.85
CUN-BAE tertiles, %		Overall		^a,d^	^f^	^c^	
		<30.41	73.9	26.3	58.8	18.6	
		30.41–39.67	26.1	30.5	38.2	36.4	
		≥39.67	0.0	43.2	2.9	44.9	
CI, mean (SD)			1.22 ± 0.12	1.29 ± 0.10 ^a,e^	1.27 ± 0.12 ^b,f^	1.33 ± 0.10 ^c^	1.30 ± 0.11
CI tertiles, %		Overall		^a,e^	^f^	^c^	
		<1.26	59.1	39.0	44.1	21.1	
		1.26–1.35	26.1	28.8	26.5	34.4	
		≥1.35	14.8	32.2	29.4	44.5	
ABSI, mean (SD)			0.080 ± 0.007	0.080 ± 0.006 ^e^	0.082 ± 0.007 ^b^	0.082 ± 0.006 ^c^	0.081 ± 0.006
ABSI tertiles, %		Overall				^c^	
		<0.079	47.7	41.5	29.4	30.0	
		0.079–0.083	18.2	21.2	23.5	25.5	
		≥0.083	34.1	37.3	47.1	44.5	
CNR1 gene polymorphisms	rs806368 (Heterozygous and Mutant), %		44.9	39.2	41.4	44.1	42.9
	rs754387 (Heterozygous and Mutant), %		60.9	57.8	46.4	60.2	58.7

ISNW Insulin-Sensitive Normal Weight, IRNW Insulin-Resistant Normal Weight, ISO Insulin-Sensitive Obesity, IRO Insulin-Resistant Obesity, HOMA-IR homeostasis model assessment for insulin resistance, VAI visceral adiposity index, LAP lipid accumulation product, CUN-BAE adiposity estimator—Clinical University of Navarra, CI conicity index, ABSI a body shape index, CNR1 cannabinoid receptor 1; ^a^ *p* < 0.05 for ISO vs. ISNW; ^b^ *p* < 0.05 for IRNW vs. ISNW; ^c^ *p* < 0.05 for IRO vs. ISNW; ^d^ *p* < 0.05 for IRNW vs. ISO; ^e^ *p* < 0.05 for IRO vs. ISO; ^f^ *p* < 0.05 for IRO vs. IRNW.

**Table 4:** Adipocytokines, insulin resistance index, and CNR1 gene polymorphism according to obesity metabolic phenotypes.

Parameters	ISO	IRNW	IRO
	OR (95%CI)	OR (95%CI)	OR (95%CI)
Age (years)	0.94 (0.89–0.99) *	0.90 (0.84–0.96) *	0.9 (0.84–0.95) *
TC (mg/dL)	1.00 (0.99–1.02)	1.00 (0.99–1.02)	1.00 (0.98–1.02)
TG (mg/dL)	1.03 (1–1.07)	1.00 (0.98–1.03)	1.02 (0.98–1.06)
HDL cholesterol (mg/dL)	0.98 (0.93–1.04)	1.01 (0.96–1.07)	0.97 (0.91–1.03)
Adiponectin (ng/mL)	0.98 (0.9–1.06)	1.03 (0.98–1.08)	0.98 (0.9–1.06)
Leptin (ng/mL)	0.97 (0.9–1.05)	1.00 (0.94–1.06)	0.99 (0.92–1.07)
VAI	0.92 (0.36–2.37)	1.3 (0.57–2.97)	0.86 (0.35–2.15)
LAP	0.91 (0.82–1.02)	0.98 (0.9–1.06)	0.95 (0.85–1.06)
CUN-BAE	1.54 (1.29–1.83) *	1.15 (1.01–1.31) *	1.51 (1.26–1.8) *
CNR1 rs754387 (heterozygous and mutant)	1.21 (0.36–4.04)	0.56 (0.17–1.88)	1.4 (0.42–4.69)
CNR1 rs806368 (heterozygous and mutant)	0.73 (0.22–2.4)	0.48 (0.14–1.61)	0.8 (0.24–2.65)
Prediabetes	2.46 (0.42–14.24)	10.75 (1.82–63.62) *	11.58 (1.88–71.15) *
Diabetes	0.81 (0.18–3.65)	3.04 (0.62–14.85)	2.67 (0.56–12.81)
Abdominal obesity	0.71 (0.19–2.6)	0.46 (0.11–1.93)	0.68 (0.18–2.48)
CI second tertiles (1.26–1.35)	1.94 (0.05–73.23)	0.42 (0.03–5.81)	3.39 (0.08–139.32)
CI third tertiles ≥ 1.35	0.54 (0.01–36.28)	0.8 (0.03–25.51)	1.16 (0.02–86.79)
ABSI second tertiles (0.079–0.083)	0.01 (0.01–0.51) *	13.42 (0.96–188.18)	0.01 (0.01–0.38) *
ABSI third tertiles ≥ 0.083	0.02 (0.01–1.59)	36.04 (1.08–1207.55) *	0.01 (0.01–0.81) *

ISNW Insulin-Sensitive Normal Weight, IRNW Insulin-Resistant Normal Weight, ISO Insulin-Sensitive Obesity, IRO Insulin-Resistant Obesity, HOMA-IR homeostasis model assessment for insulin resistance, VAI visceral adiposity index, LAP lipid accumulation product, CUN-BAE adiposity estimator—Clinical University of Navarra, CI conicity index, ABSI a body shape index, CNR1 cannabinoid receptor 1; * *p* < 0.05, OR odd ratio, CI confidence interval. The regression analysis was adjusted for covariates (sex, smoking). ISNW was considered reference category.

## Data Availability

The raw data supporting the conclusions of this article will be made available by the authors on request due to privacy.

## Abbreviations

The following abbreviations are used in this manuscript:

BMI	Body Mass Index
IRNW	Insulin-Resistant Normal Weight
ISO	Insulin-Sensitive Obesity
ISNW	Insulin-Sensitive Normal Weight
IRO	Insulin-Resistant Obesity
HOMA-IR	Homeostasis Model Assessment for Insulin Resistance
VAI	Visceral Adiposity Index
LAP	Lipid Accumulation Product
CI	Conicity Index
ABSI	Body Shape Index
CUN-BAE	Clinica Universidad De Navarra Body Adiposity Estimator
CNR1	Cannabinoid Receptor Type 1
OGTT	Oral Glucose Tolerance Test
FPG	Fasting Plasma Glucose
NGT	Normal Glucose Tolerance
TC	Total Cholesterol
TG	Triglycerides
HDL	High-Density Lipoprotein
LDL	Low-Density Lipoprotein
SD	Standard Deviation
